# Association Between Self-rating Depression Scores and Total Ghrelin and Adipokine Serum Levels in a Large Population-Based Sample

**DOI:** 10.3389/fpsyt.2022.891325

**Published:** 2022-05-11

**Authors:** Dirk Alexander Wittekind, Jürgen Kratzsch, Ronald Biemann, Roland Mergl, Steffi Riedel-Heller, Veronika Witte, Arno Villringer, Michael Kluge

**Affiliations:** ^1^Department of Psychiatry and Psychotherapy, University of Leipzig, Leipzig, Germany; ^2^Institute of Laboratory Medicine, Clinical Chemistry and Molecular Diagnostics, University of Leipzig, Leipzig, Germany; ^3^Institute of Psychology, Universität der Bundeswehr München, Neubiberg, Germany; ^4^Faculty of Medicine, Institute of Social Medicine, Occupational Health and Public Health, University of Leipzig, Leipzig, Germany; ^5^Department of Neurology, Max Planck Institute for Cognitive and Brain Sciences, Leipzig, Germany

**Keywords:** ghrelin, leptin, adiponectin, depression, CES-D, IDS-SR

## Abstract

**Background:**

Ghrelin and the adipokines leptin and adiponectin have been suggested to be involved in mood and anxiety regulation and to be altered in affective disorders. However, studies investigating the association between ghrelin, leptin and adiponectin and depressive symptomatology are scarce but might contribute to a better understanding of their involvement in mood regulation. We thus aimed investigating the association between depressive symptomatology and total ghrelin as well as leptin and adiponectin serum levels in a large population-based sample.

**Methods:**

Total serum ghrelin, adiponectin and leptin levels were determined in 1666 subjects of a population-based cross-sectional study (“LIFE”). The Center for Epidemiological Studies Depression Scale (CES-D) and the Inventory of Depressive Symptoms – Self Rating (IDS-SR) were administered. Multiple linear regression analyses were conducted to examine the association between total serum ghrelin, leptin and adiponectin and the intensity of depressive symptoms.

**Results:**

In the total sample (*n* = 1,092), neither ghrelin nor leptin or adiponectin serum levels showed a significant association with CES-D or IDS-SR sum scores (*N* = 1,092) or in depressed/non-depressed subjects. Leptin serum levels showed a significantly positive association with IDS-SR sum scores in elderly men (≥60 years; β = 0.122, 95% CI: 0.009; 0.236; *p* = 0.035).

**Conclusion:**

Our study suggests that peripheral levels of ghrelin and adipokines in a cross-sectional study design might not be sufficient to measure their involvement in depression, suggesting that associations are more complex and multi-layered.

## Introduction

Ghrelin, a 28-amino-acid peptide, is synthesized in the stomach mucosa and is the only known peripheral orexigenic hormone (1). It has been hypothesized to be a survival hormone (2) and as such is secreted in times of stress and hunger to promote food intake, reduce energy expenditure and thus promote energy homeostasis and weight gain (3, 4). Accordingly, increasing evidence has implicated ghrelin as a hormone involved in stress coping both on a metabolic (2) and behavioral level (5). Ghrelin serum levels were reproducibly shown to be increased in both acute and chronic stress in both animals (6) and humans (7–9) and were shown to remain elevated, even after the stressor had subsided in humans (10).

Depression is a state of chronic stress (11) and its association with the Hypothalamic-Pituitary-Adrenal (HPA)-axis has been very well documented (12). As such, it seems intuitive that ghrelin serum levels should be elevated in a depressive state, either as a direct effect of the stress or indirectly as a consequence of interaction with the HPA-axis, as cortisol increases ghrelin levels and vice versa (13–15). In fact, most studies investigating ghrelin serum levels in depressed patients have shown increased acylated (16, 17) and total (18) ghrelin levels. However, data is surprisingly heterogenous with some studies showing no changes in ghrelin serum levels in depression (19–22) and one even a decrease (23). The reasons for this heterogeneity remain unclear to date.

Leptin and adiponectin are both peptide hormones synthesized in adipocytes and exert in some respects antagonistic actions to ghrelin in the brain, leading to appetite reduction, feeling of satiety and a decrease of energy intake (24, 25). Both have been linked with MDD, but in a quite inconsistent manner and dependent on different other factors, like weight and sex. Low leptin levels were linked to MDD in middle-aged adults (26, 27) while in older age, high levels were associates with depression (28, 29). No association of leptin with depressive symptomatology was described in one study (30). In general, the link between leptin and depressive symptoms seems to be more pronounced in men (31). Increased adiponectin levels have been described in sub-syndromal depression (32), but in moderate and severe depression they did not differ from healthy controls (32). On the other hand, one study found lower levels in depression (33).

Whether ghrelin, leptin and adiponectin serum levels increase as a consequence of a disease state, i.e., a depressive episode in MDD or as a consequence of depressive symptomatology which is present during a depressive episode is unclear. Only one very recent study investigated the association between ghrelin and adipokine serum levels and depressive symptomatology thus far (34). They found no association between total ghrelin, leptin and adiponektin serum levels and depressive symptomatology in a cross-sectional analysis at baseline.

We aimed at investigating the association between total ghrelin, leptin and adiponectin serum levels and depressive symptomatology, as assessed with the Center for Epidemiological Studies Depression Scale (CES-D) and the Inventory of Depressive Symptoms (IDS) in a population-based sample from the LIFE-Adult study.

## Methods

### Study Sample

Subjects were recruited in the context of the LIFE-Adult-Study (Leipzig Research Center for Civilization Diseases), a population-based cohort study with 10,000 participating adults mainly aged 40–79 years. The sample was randomly recruited in the city of Leipzig in Germany (35). Blood samples were collected after an overnight fast including abstinence from smoking between 07:30 and 10:30 h. The blood samples had been immediately processed by the team of the LIFE pre-analytical laboratory which is part of the Leipzig Medical Biobank and sent directly to the Institute of Laboratory Medicine, Clinical Chemistry, and Molecular Diagnostics (ILM) where direct analysis was carried out. Additional samples were frozen at −80°C until usage for ghrelin measurements.

Subjects were excluded if they fulfilled the following criteria: current treatment of autoimmune diseases, presence of epileptic seizures in the last year, current treatment of multiple sclerosis, current diseases of the gastrointestinal system and treatment in the last year due to a diagnosis of cancer. Total ghrelin serum concentrations were available for 1,666 participants of the LIFE study. From these, 45 individuals were excluded because they did not meet inclusion or exclusion criteria. 529 further subjects had to be excluded because their data sets were not complete regarding at least one of the following variables: age, sex, BMI, alcohol intake, smoking status, cortisol level, CES-D and GAD-7 sum scores.

Thus, the resulting study population comprised 1,092 persons (633 men and 459 women). Regarding IDS sum scores they had been examined in a subgroup of the final sample (*n* = 601).

The procedures were conducted according to the Declaration of Helsinki and approved by the Ethics Committee of the University of Leipzig (registration-number: 263-2009-14122009).

### Ghrelin, Leptin and Adiponectin Measurements

All ghrelin measurements were done in serum by the use of a radioimmunoassay for total ghrelin (Millipore, GHRT-89HK, Missouri, USA). Samples were not pre-treated with enzyme inhibitors or acidification. Due to this, only total ghrelin was measured, as it was not possible to discriminate between the two circulating isoforms, acyl- and desacyl-ghrelin. Intra-assay coefficients of variation were 3.3–10.0%; inter-assay coefficients of variation were between 14.7 and 17.8%. and detection limit was 93 pg/ml.

Serum concentrations of adiponectin were measured with a sandwich ELISA by Mediagnost GmbH (E09, Reutlingen, Germany) with intra-assay coefficients of variation between 3.1 and 3.6%, inter-assay coefficient of variation of 12% and a limit of quantification of 0.27 μg/l. Serum leptin concentrations were measured with a sandwich ELISA by Mediagnost GmbH (E07, Reutlingen, Germany) with intra-assay coefficients of variation between 4.7 and 5.2%, inter-assay coefficients of variation between 4.4 and 4.8% and a limit of detection of 0.25 μg/l.

### Center for Epidemiological Studies Depression Scale (CES-D)

The CES-D is a short self-report scale designed to assess and quantify depressive symptoms in a general population. It consists of 20 items assessing typical symptoms of depression, each one scored using a 4-point-Likert-scale (0 = rarely/almost none of the time; 3 = most or all of the time). Accordingly, the minimum score is 0, indicating the absence of depressive symptoms and the maximum score is 60, indicating strong depressive symptoms. The cut-off score for relevant depressive symptoms was defined by the authors at 16 or higher (26). We chose a higher cut-off of ≥22 (36), as discriminability was higher at this cut-off. The scale has a high internal consistency, and adequate test-retest repeatability (37).

### Inventory of Depressive Symptoms–Self Rating

The Inventory of Depressive symptoms is a self-rating questionnaire assessing all symptoms of a Major Depressive Disorder (MDD), including its subtypes. It contains 30 items ranging from 0 to 3. According to the total score, subjects can be categorized into the following subgroups: No clinically relevant depressive symptoms (IDS sum score: 0–13), mild intensity of depressive symptoms (IDS sum score: 14–25), moderate intensity of depressive symptoms (IDS sum score: 26–38), severe intensity of depressive symptoms (IDS sum score: 39–48), extreme intensity of depressive symptoms (IDS sum score: >48). The scale has a high internal consistency (Crohnbach's alpha 0.92–0.94) (38, 39). In this sample, the IDS was only performed in elderly subjects (minimum age: 60 years).

### Generalized Anxiety Disorder Scale 7

The 7- item Generalized Anxiety Disorder Scale was developed to assess the presence and severity of anxiety symptoms in a time period of 2 weeks preceding testing. It is a self-rating instrument that encompasses 7 items that can be rated from 0 (not at all) to 3 (nearly every day). Thus, the maximum score is 21 and the cut-off for clinically relevant anxiety symptoms is ≥10 points. It has a high internal consistency of 0.92 and re-test reliability (intraclass correlation of 0.83) (37).

### Acquisition of Data on Tobacco and Alcohol Consumption

Tobacco consumption was assessed solely via self-administered questionnaire and interview. No biological verification method was used (like measurement of carbon monoxide in breath). Subjects were grouped in three categories: active smoker, former smoker and never-smoker. Active smokers were considered all those participants who had smoked regularly for at least 6 months consecutively in their lifetime and at least occasionally at the time of examination. Subjects who had smoked continuously for more than 6 months during their lifetime, but were not smoking at the time of assessment, were defined as former smokers. Cigarette equivalents (number per day) were calculated as follows: One cigarette equivalent was defined as 1 g of tobacco, 1 cigar was defined as 4 g of tobacco (i.e., four cigarette equivalents), 1 pipe as 3 g of tobacco (i.e., 3 cigarette equivalents), 1 cigarillo as 2 g of tobacco (i.e., 2 cigarette equivalents). For further information see Latza et al. (40).

Frequency and amount of consumption of alcoholic beverages (i.e., beer/wine/spirits) during the last 12 months were semiquantitatively assessed using a self-administered food frequency and alcohol questionnaire (FFQ). Possible answers for the frequency of alcohol consumption were “multiple times a day”, “daily”, “multiple times a week”, “once a week”, “two to three times a month”, “once a month or rarer” or “almost never”. Also, the amount of beverage consumption was assessed by defined categories. From the amount and frequency of alcoholic beverage as well as the average alcohol content of different beverages, the average consumption of pure alcohol (g/day) was calculated.

### Assessment of BMI

The BMI is defined as the body weight divided by the square of the body height (kg/m2). Body weight was measured with an electronic scale (SECA 701, Seca GmbH & Co KG) with a precision of 0.01 kg, height using a stadiometer (SECA 240) to the nearest 0.1 cm by trained staff according to standardized protocols.

### Statistical Analysis

Multivariate linear regression analyses were calculated for both the total sample and two subgroups [depressed individuals (CES-D sum score ≥ 22 and IDS sum score ≥ 14, respectively) and participants without clinically relevant depressive symptoms (CES-D sum score <22 and IDS sum score <14, respectively)]. The independent variable was the ghrelin or leptin or adiponectin concentration and the dependent variables were the CES-D and IDS sum scores. The analyses were adjusted for age, sex (reference category: male) (3), smoking status (reference category: non-smokers) (41), alcohol consumption (42), BMI scores (3), cortisol levels (15) and GAD-7 (43) sum scores, which were recently shown to be associated with ghrelin serum levels (43). Regression coefficients (β) with 95% confidence intervals (CI) were calculated. For post-hoc analyses in men and women, the same covariables were chosen (without sex.

For additional analyses, we built nine new variables in order to assess their association with ghrelin, leptin and adipokine in a next step:

IDS hyposomnia: sum of the IDS items 1–3

➢ IDS hypersomnia: IDS item 4;
➢ IDS hypo-appetite: IDS item 11;
➢ IDS hyper-appetite: IDS item 12;
➢ IDS decrease of weight: IDS item 13;
➢ IDS increase of weight: IDS item 14;
➢ CES-D sleep disturbances: sum of the CES-D items 5, 11 and 17;
➢ CES-D appetite disturbances: CES-D item 1;
➢ CES-D changes of weight: CES-D item 16.

The association of these variables with ghrelin, leptin and adipokine was assessed by using 27 multiple linear regression analyses with the dependent variables being the nine afore-mentioned IDS and CES-D sub-scores. For each sub-score as dependent variable, three regression analyses were computed with the independent variables being 1. ghrelin, 2. leptin and 3. adipokine. The covariables were in all cases: age, sex, BMI score, alcohol consumption, smoking status, cortisol concentration, GAD-7 sum score.

The SPSS software (version 26.0) was used for the statistical analyses. The significance level was set at α = 0.05. All statistical tests were two-tailed.

## Results

### Clinical Data

The study sample was described in Table 1. Overall, the intensity of depressive symptoms was low in the total sample (mean CES-D sum score: 9.23, standard deviation (S.D.): 6.07; mean IDS sum score: 8.94; S.D.: 6.20), with 50 persons (4.58%) showing depressive symptoms in clinically relevant intensity (CES-D sum scores: 22-60) (see Supplementary Table S1). The corresponding percentage of elderly subjects (minimum age: 60 years) based on an IDS cut-off sum score of ≥ 14 points was 19.47% (117 of 601 individuals with valid IDS sum scores).

**Table 1:** Description of the sample.

**Variable**	**Total sample** **(n = 1,092)**	**Males** **(n = 633) (57.97%)**	**Females** **(n = 459) (42.03%)**	***p*-value**
Ghrelin serum concentration, pg/ml, mean (SD)	907.29 (441.96)	805.48 (314.04)	1,047.68 (543.26)	**<0.001** ^***^ ^a^
Adiponectin serum concentration, ng/l, mean (SD)	7,185.72 (4,451.57)	5,505.60 (3,030.14)	9,502.75 (5,025.23)	**<0.001** ^***^ ^a^
Leptin serum concentration, μg/l, mean (SD)	11.22 (11.69)	6.21 (5.96)	18.13 (13.94)	**<0.001** ^***^ ^a^
Age, years, mean (SD)	57.07 (16.26)	57.34 (16.49)	56.70 (15.95)	0.18^a^
BMI, kg/m^2^, mean (SD)	26.99 (4.48)	27.27 (4.04)	26.60 (5.01)	**<0.001** ^***^ ^a^
Alcohol consumption, g/day, mean (SD)	12.99 (17.76)	18.29 (20.30)	5.69 (9.54)	**<0.001** ^***^ ^a^
Non-smokers (%)	941 (86.2%)	531 (83.9%)	410 (89.3%)	0.0102 ^*^^b^
Active smokers (%)	151 (13.8%)	102 (16.1%)	49 (10.7%)	—
Cortisol concentration, nmol/l, mean (SD)	512.62 (196.35)	496.38 (145.01)	535.02 (248.91)	0.85^a^
GAD-7 sum score, mean (SD)	2.69 (2.70)	2.44 (2.60)	3.05 (2.81)	<0.001^***^^a^
CES-D sum score, mean (SD)	9.23 (6.07)	8.59 (5.26)	10.11 (6.94)	**0.0011** **^a^
IDS sum score, mean (SD)	8.94 (6.20) (*n* = 601)	7.92 (5.61) (*n* = 353)	10.40 (6.69) (*n* = 248)	**<0.001** ^***^ ^a^

*BMI, Body Mass Index; CES-D, Center for Epidemiological Studies Depression Scale (44, 45); CI, confidence interval; GAD-7, Generalized Anxiety Disorder 7-item Scale (37, 46); SD, Standard Deviation*.

* p ≤ 0.05; **p ≤ 0.01;

*** *p ≤ 0.001*.

a *Due to non-normal distribution of the dependent variables (Kolmogorov-Smirnov-Test: p <0.05), a Mann-Whitney U test was performed in order to compute p values regarding ghrelin serum concentrations (Z = 0.149), age (Z = 0.181), BMI scores (Z = 0.071), alcohol consumption (Z = 0.233), cortisol concentration (Z = 0.076), GAD-7 sum scores (Z = 0.163), CES-D sum scores (Z = 0.114) and IDS sum scores (Z = 0.149), adiponectin concentrations (Z = −15.522) and leptin concentrations (Z = −19.478)*.

b *A chi^2^ test for a two-by-two cross table (χ^2^ = 6.60; df = 1) was applied.*

*Results with a p-values > 0.05 are marked bold*.

### Association Between the Severity of Depressive Symptoms and Ghrelin Serum Levels

In the total sample (*N* = 1,092), ghrelin serum levels did not show a significant association with CES-D sum scores when adjusted for age, sex, BMI, alcohol consumption, smoking status, cortisol concentrations and GAD-7 sum scores (ß = −0.00033, standardized β = −0.024, 95% CI: −0.0010; 0.00039; p = 0.366) (see Table 2, upper part). Regarding depressed individuals (CES-D sum score ≥ 22), the association between ghrelin serum levels and CES-D sum scores was not significant, too (ß = −0.00079, standardized β = −0.077, 95% CI: −0.0039;0.0023; p = 0.605; adjusted for the above mentioned covariables) (see Table 2, middle part). For persons without clinically relevant depressive symptoms (CES-D sum score <22), ghrelin serum levels were also not significantly associated with CES-D sum scores with the effects of the above listed covariables being controlled for (β = −0.0004; standardized β = −0.038; 95% CI: −0.0010; 0.00022; *p* = 0.206) (see Table 2, lower part).

**Table 2:** Results of multiple linear regression analyses regarding the association between ghrelin serum levels and CES-D sum scores reflecting the intensity of depressive symptoms in participants of the study.

**Variables**	**Regression coefficient β (95% CI)**	**Standardized ß**	**t**	***p* value**
**CES-D sum scores in the total sample (*****N*** **=** **1,092)**				
Corrected R^2^ = 0.319; F = 64.96; p < 0.001^***^				
Multivariate model^a^	—	—	—	—
Ghrelin serum levels	−0.00033 (−0.0010;0.00039)	−0.024	−0.904	0.366
Age	0.002 (−0.019;0.022)	0.004	0.150	0.881
Sex	1.014 (0.335;1.694)	0.083	2.928	0.003^**^
Alcohol consumption	0.006 (−0.012;0.024)	0.018	0.654	0.513
Smoking Status	0.243 (−0.188;0.675)	0.029	1.106	0.269
BMI	0.099 (0.026;0.173)	0.073	2.661	0.008^**^
Cortisol concentration	0.001 (−0.001;0.002)	0.022	0.812	0.417
GAD-7 sum score	1.247 (1.136;1.358)	0.556	21.975	<0.001^***^
**CES-D sum scores in the subgroup of individuals with clinically relevant intensity of depressive symptoms (CES-D sum scores** **≥22) (*****n*** **=** **50)**				
**Corrected R**^**2**^ **=** **0.044; F** **=** **1.283; p** **=** **0.279**				
Multivariate model^a^	—	—	—	—
Ghrelin serum levels	−0.00079 (−0.0039;0.0023)	−0.077	−0.521	0.605
Age	−0.115 (−0.227;-0.002)	−0.340	−2.053	0.046^*^
Sex	1.942 (−2.750;6.635)	0.151	0.836	0.408
Alcohol consumption	0.129 (-0.011;0.268)	0.317	1.865	0.069^+^
Smoking Status	−1.156 (−3.940;1.628)	−0.137	−0.839	0.407
BMI	0.023 (−0.404;0.450)	0.017	0.110	0.913
Cortisol concentration	−0.002 (−0.009;0.006)	−0.063	−0.398	0.693
GAD-7 sum score	0.344 (−0.211;0.898)	0.185	1.252	0.218
**CES-D sum scores in the subgroup of individuals without clinically relevant intensity of depressive symptoms (CES-D sum scores** **<** **22) (*****n*** **=** **1,042)**				
**Corrected R**^**2**^ **=** **0.166; F** **=** **26.89;** ***p*** **<** 0.001^***^				
Multivariate model^a^	—	—	—	—
Ghrelin serum levels	−0.0004 (−0.0010;0.0002)	−0.038	−1.266	0.206
Age	0.021 (0.004;0.038)	0.076	2.458	0.014^*^
Sex	0.459 (−0.110;1.029)	0.050	1.583	0.114
Alcohol consumption	0.001 (−0.015;0.016)	0.002	0.072	0.943
Smoking Status	0.302 (−0.059;0.663)	0.049	1.643	0.101
BMI	0.068 (0.007;0.130)	0.068	2.188	0.029^*^
Cortisol concentration	0.001 (−0.00003;0.003)	0.058	1.922	0.055^+^
GAD-7 sum score	0.756 (0.649;0.863)	0.396	13.858	<0.001^***^

*b, Regression coefficient; β, Standardized regression coefficient; BMI, Body Mass Index; CES-D, Center for Epidemiological Studies Depression Scale (44, 45); CI, confidence interval; GAD-7, Generalized Anxiety Disorder 7-item Scale (37, 46); N/n: sample sizes.*

+ *p ≤ 0.10;*

* *p ≤ 0.05;*

** *p ≤ 0.01;*

*** *p ≤ 0.001.*

a *All multivariate linear regression models have been adjusted for age, Sex, alcohol consumption, smoking status (1 = active smoking; 0 = non-smoking), BMI scores, cortisol concentrations and GAD-7 sum scores.*

*Results with a p-values > 0.05 are marked bold*.

Clinically relevant depressive symptoms were detected by the CES-D in 4.58% of the study sample. This in line with previous epidemiological data in similar populations (47).

Similar results were present if the intensity of depressive symptoms was measured by using IDS sum scores (see Supplementary Table S2).

### Association Between the Severity of Depressive Symptoms and Adiponectin Serum Levels

In the total sample, adiponectin serum levels were not significantly associated with CES-D sum scores when adjusted for the afore-mentioned covariables (ß = 0.00006, standardized β = 0.044, 95% CI: −0.00002; 0.0001; p = 0.132 (see **Table 4**, upper part). Regarding depressed individuals (CES-D sum score ≥ 22), the association between adiponectin serum levels and CES-D sum scores failed to be significant, too (ß = −0.0001, standardized β = −0.097, 95% CI: −0.0005;0.0003; *p* = 0.573) (see Table 3, middle part). For individuals without clinically relevant depressive symptoms (CES-D sum score <22), adiponectin serum levels were also not significantly associated with CES-D sum scores (β = 0.00004; standardized β = 0.036; 95% CI: −0.00003;0.0001; *p* = 0.276) (see Table 3, lower part).

**Table 3:** Results of multiple linear regression analyses regarding the association between adiponectin serum levels and CES-D sum scores reflecting the intensity of depressive symptoms in participants of the study.

**Variables**	**Regression coefficient β (95% CI)**	**Standardized ß**	**t**	**p value**
**CES-D sum scores in the total sample (*****N*** **=** **1,092)**				
**Corrected R**^**2**^ **=** **0.320; F** **=** **65.232;** ***p*** **<** 0.0001^***^				
Multivariate model^a^	—	—	—	—
Adiponectin serum levels	0.00006 (-0.000018;0.000138)	0.044	1.506	0.132
Age	−0.002 (-0.023;0.018)	−0.007	−0.231	0.817
Sex	0.703 (-0.017;1.422)	0.057	1.917	0.055
Alcohol consumption	0.006 (-0.012;0.024)	0.017	0.615	0.538
Smoking Status	0.248 (-0.183;0.680)	0.029	1.129	0.259
BMI	0.119 (0.046;0.193)	0.088	3.182	0.002^**^
Cortisol concentration	0.001 (-0.001;0.002)	0.023	0.874	0.382
GAD-7 sum score	1.243 (1.132;1.354)	0.554	21.966	<0.001^***^
**CES-D sum scores in the subgroup of individuals with clinically relevant intensity of depressive symptoms (CES-D sum scores** **≥22) (*****n*** **=** **50)**				
**Corrected R**^**2**^ **=** **0.045; F** **=** **1.291;** ***p*** **=** **0.275**				
Multivariate model^a^	—	—	—	—
Adiponectin serum levels	−0.00011 (−0.001;0.00029)	−0.097	−0.568	0.573
Age	−0.105 (−0.225;0.015)	−0.311	−1.759	0.086
Sex	1.961 (-2.730;6.651)	0.153	0.844	0.403
Alcohol consumption	0.125 (-0.013;0.262)	0.306	1.831	0.074
Smoking Status	−1.309 (−4.188;1.570)	−0.155	−0.919	0.364
BMI	0.014 (−0.418;0.447)	0.011	0.068	0.946
Cortisol concentration	−0.001 (−0.009;0.007)	−0.031	−0.190	0.850
GAD-7 sum score	0.339 (−0.216;0.893)	0.183	1.234	0.224
**CES-D sum scores in the subgroup of individuals without clinically relevant intensity of depressive symptoms (CES-D sum scores** **<** **22) (*****n*** **=** **1,042)**				
**Corrected R**^**2**^ **=** **0.166; F** **=** **26.829; p** **<** 0.0001^***^				
Multivariate model^a^	—	—	—	—
Adiponectin serum levels	0.000037 (−0.000029;0.000103)	0.036	1.091	0.276
Age	0.019 (0.001;0.036)	0.067	2.106	0.035^*^
Sex	0.219 (-0.386;0.824)	0.024	0.711	0.477
Alcohol consumption	0.000188 (-0.015;0.015)	0.001	0.024	0.980
Smoking Status	0.296 (-0.065;0.657)	0.048	1.610	0.108
BMI	0.085 (0.023;0.146)	0.085	2.688	0.007^**^
Cortisol concentration	0.001 (0.000027;0.003)	0.061	2.001	0.046^*^
GAD-7 sum score	0.753 (0.646;0.860)	0.394	13.821	<0.001^***^

*b, Regression coefficient; β, Standardized regression coefficient; BMI, Body Mass Index; CES-D, Center for Epidemiological Studies Depression Scale (44, 45); CI, confidence interval; GAD-7, Generalized Anxiety Disorder 7-item Scale (37, 46); N/n: sample sizes.*

*^+^p ≤ 0.10;*

* *p ≤ 0.05;*

** *p ≤ 0.01;*

** *p ≤ 0.001.*

a *All multivariate linear regression models have been adjusted for age, sex, alcohol consumption, smoking status (1 = active smoking; 0 = non-smoking), BMI scores, cortisol concentrations and GAD-7 sum scores.*

*Results with a p-values > 0.05 are marked bold*.

Similar results were obtained if the intensity of depressive symptoms was measured with IDS sum scores (see Supplementary Table S2).

### Association Between the Severity of Depressive Symptoms and Leptin Serum Levels

In the total sample, leptin serum levels were not significantly associated with CES-D sum scores when adjusted for age, sex, BMI, alcohol consumption, smoking status, cortisol concentrations and GAD-7 sum scores (ß = 0.013, standardized β = 0.024, 95% CI: −0.031; 0.056; *p* = 0.569 (see Table 4, upper part). The same was true for depressed individuals (CES-D sum score ≥ 22) (ß = 0.215, standardized β = 0.405, 95% CI:−0.017;0.446; *p* = 0.068) (see Table 4, middle part) and for individuals without clinically relevant depressive symptoms (CES-D sum score <22) (β = 0.002; standardized β = 0.006; 95% CI: −0.034;0.039; *p* = 0.903) (see Table 4, lower part).

**Table 4:** Results of multiple linear regression analyses regarding the association between leptin serum levels and CES-D sum scores reflecting the intensity of depressive symptoms in participants of the study.

**Variables**	**Regression coefficient β (95% CI)**	**Standardized ß**	**t**	***p* value**
**CES–D sum scores in the total sample (*****N*** **=** **1,092)**				
**Corrected R**^**2**^ **=** **0.319; F** **=** **64.873;** ***p*** **<** 0.0001^***^				
Multivariate model^a^	——	——	——	——
Leptin serum levels	0.013 (−0.031;0.056)	0.024	0.570	0.569
Age	0.002 (−0.018;0.022)	0.005	0.173	0.863
Sex	0.771 (−0.085;1.628)	0.063	1.767	0.078
Alcohol consumption	0.006 (−0.012;0.024)	0.017	0.625	0.532
Smoking Status	0.236 (−0.196;0.667)	0.028	1.071	0.284
BMI	0.085 (−0.019;0.188)	0.063	1.609	0.108
Cortisol concentration	0.001 (−0.001;0.002)	0.022	0.832	0.406
GAD−7 sum score	1.243 (1.132;1.354)	0.554	21.943	<0.001^***^
**CES–D sum scores in the subgroup of individuals with clinically relevant intensity of depressive symptoms (CES–D sum scores** **≥22) (*****n*** **=** **50)**				
**Corrected R**^**2**^ **=** **0.114; F** **=** **1.785;** ***p*** **=** **0.108**				
Multivariate model^a^	——	——	——	——
Leptin serum levels	0.215 (−0.017;0.446)	0.405	1.872	0.068
Age	−0.092 (−0.204;0.019)	−0.274	−1.672	0.102
Sex	−1.677 (−7.486;4.132)	−0.130	−0.583	0.563
Alcohol consumption	0.107 (−0.026;0.240)	0.264	1.626	0.112
Smoking Status	−1.264 (−3.941;1.412)	−0.150	−0.954	0.346
BMI	−0.288 (−0.828;0.252)	−0.214	−1.077	0.288
Cortisol concentration	−0.001 (−0.008;0.007)	−0.031	−0.203	0.840
GAD−7 sum score	0.305 (−0.231;0.840)	0.164	1.149	0.257
**CES–D sum scores in the subgroup of individuals without clinically relevant intensity of depressive symptoms (CES–D sum scores** **<** **22) (*****n*** **=** **1,042)**				
**Corrected R**^**2**^ **=** **0.165; F** **=** **26.652;** ***p*** **<** 0.0001^***^				
Multivariate model^a^	——	——	——	——
Leptin serum levels	0.002 (−0.034;0.039)	0.006	0.122	0.903
Age	0.021 (0.004;0.038)	0.075	2.407	0.016^*^
Sex	0.332 (−0.385;1.049)	0.036	0.909	0.364
Alcohol consumption	0.0002 (−0.015;0.015)	0.001	0.021	0.983
Smoking Status	0.289 (−0.072;0.650)	0.046	1.573	0.116
BMI	0.073 (−0.014;0.160)	0.073	1.641	0.101
Cortisol concentration	0.001 (0.000003;0.003)	0.060	1.966	0.05^*^
GAD−7 sum score	0.752 (0.645;0.859)	0.393	13.794	<0.001^***^

*b, Regression coefficient; β, Standardized regression coefficient; BMI, Body Mass Index; CES-D, Center for Epidemiological Studies Depression Scale (44, 45); CI, confidence interval; GAD-7, Generalized Anxiety Disorder 7-item Scale (37, 46); N/n: sample sizes.*

*^+^p ≤ 0.10;*

* p ≤ 0.05; **p ≤ 0.01;

*** *p ≤ 0.001.*

a *All multivariate linear regression models have been adjusted for age, sex, alcohol consumption, smoking status (1 = active smoking; 0 = non-smoking), BMI scores, cortisol concentrations and GAD-7 sum scores.*

*Results with a p-values > 0.05 are marked bold*.

However, for elderly subjects (minimum age: 60 years) a significantly positive association of leptin serum levels and intensity of depressive symptoms as measured by using IDS sum scores (ß = 0.055, standardized β = 0.108, 95% CI: 0.0003;0.109; p = 0.049; see Supplementary Table S4). *Post-hoc* analyses revealed that this finding was due to a significantly positive association between these variables in elderly men (ß = 0.122, standardized β = 0.126, 95% CI: 0.009;0.236; p = 0.035), with the corresponding associating in elderly women not being significant (ß = 0.061, standardized β = 0.134, 95% CI: −0.017;0.139; *p* = 0.127).

### Association Between Ghrelin, Leptin and Adiponectin Levels and Depression Scale Sub-scores

In 25 of 27 cases associations between ghrelin, leptin and adiponectin levels and depression scale sub-scores reflecting changes of weight or appetite and sleep disturbances failed to be statistically significant (see Supplementary Table S5).

In two cases significant findings were present: A significantly positive association between ghrelin and IDS hypo-appetite (b = 0.00007; *p* = 0.029) and a significantly positive association between adiponectin and IDS hypo-appetite (b = 0.000008; *p* = 0.033).

## Discussion

In this large, population-based sample, we investigated the association between total ghrelin, leptin and adiponectin serum levels with depressive symptomatology. We neither found a significant association between total scores in the self-rating depression questionnaires CES-D and IDS and ghrelin and adiponectin serum levels, nor did any subgroup analysis, i.e., only analyzing depressed/non-depressed, subjects reveal any significant association. For leptin, no significant association was found with CES-D scores but a significant positive association with IDS scores. The latter finding was found in elderly men, but not in elderly women. When analyzing sub-scales of the CES-D and IDS, there was a weakly significant association between ghrelin and adiponectin serum levels and reduced appetite in the IDS.

Available literature concerning ghrelin levels in MDD is relatively heterogenic with more evidence pointing to an increase in ghrelin serum levels in MDD. This has been shown in several studies (18, 48–51). Furthermore, ghrelin has been shown to cause mood elevation after acute administration (52) and first evidence points to antidepressant effects after acute administration (53). Recently, it has even been suggested, that ghrelin levels may aid in defining subtypes of depression, as MDD patients with reduced appetite had increased ghrelin while patients with increased appetite had decreased ghrelin levels (54). One study points to higher ghrelin levels in severe vs. moderate depression (17). However, other studies have found unaltered (19, 55, 56) or even decreased (23) ghrelin levels in MDD patients as compared to healthy controls.

These heterogenic results might reflect ghrelin's complex involvement in mood, anxiety and stress regulation that recent literature has increasingly shown (5, 8). Gathering evidence from animal studies suggests that ghrelin does not have a stable antidepressive or depressiogenic effect, but that it rather depends on several circumstances which effect ghrelin exerts. These circumstances include acute vs. chronic stress exposure (6, 57–59), feeding state (60) and whether animals were stressed or unstressed previous to ghrelin exposure (57). Thus, peripheral hormone levels might not be an adequate representation of the complexity of this regulatory system.

Up until very recently, no study existed investigating the association between depressive symptomatology and ghrelin serum levels in a population-based approach. In 2022, van Andel et al. published the first study with this premise. They investigated the relationship of ghrelin serum levels and depressive symptomatology as assessed with the CES-D in a cross-sectional manner at two time points 3 years apart (34). As in our study, no significant associations were found at baseline assessment between ghrelin levels and CES-D scores. However, the authors found that higher baseline ghrelin serum levels were associated with higher CES-D scores after 3 years. This effect was only significant in younger and lean participants (34). Our results are in line with these findings insofar that we too did not find a significant association between depressive symptoms and ghrelin serum levels. Taking together this data from two recent studies with large sample sizes and similar designs strongly suggests that there seems to be no baseline, cross-sectional association between ghrelin serum levels and depressive symptomatology. This conjecture is further corroborated by another very recent study, which found no association between ghrelin serum levels and depressive symptoms at baseline in a sample of depressive patients (61). However, in this study, high ghrelin levels at baseline were predictive of a better response to anti-depressant therapy at 12 weeks.

For leptin, no association was found with CES-D scores. However, in the IDS, which was performed in older adults, an, albeit weak, significant association was found. This association got clearer when only examining men. This finding is in line with results from previous studies where the link between leptin and MDD was reproducibly strongest in older men (28, 29, 31, 62). When interpreting these results, it has to be kept in mind that leptin levels are known to be reduced in elderly women when compared to elderly men (63). Thus, the often-described association between depression scores and leptin levels seen in elderly men might be due to this phenomenon. Van Andel et al. found no significant association between leptin and depressive symptomatology at baseline, only at the three-year follow up (31). However, they only investigated the CES-D, where we also found no association, and not the IDS, as in our sample. Thus, the differing results might be due to the different item used and the fact that depression was much more prevalent in the elderly tested in the IDS than in the CES-D, which was performed in the total sample. Recent results suggest that central leptin resistance seems to play a role in MDD, which is known to progress in old age (31, 64). In general, leptin's involvement in the pathophysiology of depression is quite well documented: it decreases anxiety- and despair- behavior in mice (65–68), most likely by acting on mesolimbic neurons (69, 70). Furthermore, it has been shown to enhance SSRI-action (71, 72). A leptin analog, metreleptin, even exhibited swift antidepressant action in depressed anorexia-nervosa patients, which is associated with low leptin levels (65, 73). In a recent study, leptin was present in a proteomic cluster which was significantly associated with depressive symptoms, along with C-reactive protein (CRP) (74). In line with this, CRP inhibits leptin from binding to its receptor in mice (75), again implicating central leptin resistance as a possible pathological mechanism for depression. Much like in ghrelin, this involvement does not seem to be measurable in serum levels in a reliable and persistent manner.

Ample evidence points to an involvement of adiponectin in the pathophysiology of MDD. In humans, low adiponectin levels at baseline predicted a good response to ketamine in MDD patients (76). Furthermore, a very recent study showed that hippocampus metabolism was negatively associated with serum adiponectin levels in MDD patients, but positively associated in healthy controls (77). Regarding changes in serum levels of adiponectin in depressed patients, data is quite scarce and results have been heterogenous. Jeong and colleagues showed no association of adiponectin in moderate and severe MDD, but increased adiponectin levels in subsyndromal depression (32). Other studies investigating the association of MDD with serum adiponectin levels both found lower levels in MDD patients, one in elderly patients (33) and one in premenopausal women (78), possibly suggesting an age effect. However, the recent study by van Andel found no association of depressive symptoms with adiponectin levels in a community-based sample (31). Paralleling findings in leptin and ghrelin, adiponectin's involvement in depression pathophysiology might not be adequately reflected in serum levels.

In our sample, a striking difference was apparent in the prevalence of depressive symptomatology between the total sample (4.58%) and subjects aged 60 years or older (19.47%). This is even slightly higher than the prevalence described in the literature of around 13.5% (79). The prevalence as assessed by the CES-D lay in the

In the expected range of around 5%, which is described in many previous studies (80, 81). This highlights the high relevance of depressive symptoms in the elderly population.

The association of ghrelin and adiponectin with decreased appetite in the IDS is physiologically highly plausible, although p-values are just barely below.05 and thus these results should be interpreted with caution. Furthermore, due to the cross-sectional design of our study, a causal conclusion cannot be drawn. However, it seems likely that ghrelin levels rise due to decreased food intake in patients with decreased appetite.

Limitations of the study were the cross-sectional design, the fact that depressive symptomatology was assessed via self-rating instruments and that only total ghrelin was assessed. Also, blood samples were not pre-treated with protease inhibitors or acidification. Hence, it is not possible to discriminate between the two circulating isoforms, acyl- and desacyl-ghrelin. While the correlation between results of self-report and clinician-administered depression scales are quite high (82), for a complete assessment of depressive symptomatology, a combination of both self-rating and clinician-administered depression scales should be used (83).

Furthermore, the prevalence of clinically relevant depressive symptoms was low in the CES-D score, leading to very unbalanced group sizes. This is a common problem in epidemiological studies. Thus, interpretation of our data should be done with according caution, as it is possible that significant associations would be found in larger study samples.

Thus, for future studies, the goal should be to assess both acyl and des-acyl ghrelin, to use clinician-administered scales and to aim for even larger sample sizes to avoid under-powering of the study.

The main strengths of the studies were the large sample size and highly standardized testing and data acquisition.

In conclusion, our study provides further evidence from a well-defined sample of population-based subjects, that depressive symptomatology does not seem to be associated with total ghrelin and adiponectin serum levels and only associated with leptin levels in the sub-group of older men. This shows that even though convincing evidence shows ghrelin, leptin and adiponectin to be involved in the pathophysiology of depression and depressive symptoms, this involvement does not necessarily manifest itself in altered serum levels. This is a relevant finding that adds to our understanding of ghrelin's role in depression and mood regulation and should be taken into consideration in future studies.

## Data Availability Statement

The raw data supporting the conclusions of this article will be made available by the authors, without undue reservation.

## Ethics Statement

The studies involving human participants were reviewed and approved by Ethics Committee of the University of Leipzig (registration-number: 263-2009-14122009). The patients/participants provided their written informed consent to participate in this study.

## Author Contributions

DW: conceptualization and design, validation, project administration, and writing-original draft. JK and RB: investigation and writing–review and editing. RM: validation and writing–review and editing. SR-H: investigation, resources, and writing–review and editing. VW: funding acquisition, resources, and writing–review and editing. AV: funding acquisition and project Administration. MK: conceptualization and design, validation, project administration, writing-original draft, and supervision. All authors contributed to the article and approved the submitted version.

## Funding

This work was supported by LIFE - Leipzig Research Center for Civilization Diseases, University of Leipzig. LIFE is funded by means of the European Union, by means of the European Social Fund (ESF), by the European Regional Development Fund (ERDF), and by means of the Free State of Saxony within the framework of the excellence initiative. Part of the work was funded within the framework of the Collaborative Research Center Adiposity Mechanisms 209933838/Deutsche Forschungsgemeinschaft (DFG, German Research Foundation).

## Conflict of Interest

The authors declare that the research was conducted in the absence of any commercial or financial relationships that could be construed as a potential conflict of interest.

## Publisher's Note

All claims expressed in this article are solely those of the authors and do not necessarily represent those of their affiliated organizations, or those of the publisher, the editors and the reviewers. Any product that may be evaluated in this article, or claim that may be made by its manufacturer, is not guaranteed or endorsed by the publisher.
