# Supplementary material for: Association Between Self-rating Depression Scores and Total Ghrelin and Adipokine Serum Levels in a Large Population-Based Sample
Source: Front Psychiatry. 2022 May 11;13:891325. doi: 10.3389/fpsyt.2022.891325 (PMC9130496; doi:10.3389/fpsyt.2022.891325)
Supplement: Supplementary file 1 [file Data_Sheet_1.docx]

**Appendix:**

**Table S1: Severity distribution of depressive symptomatology**

| **Range** | **Frequency**  **(%)** | **Ghrelin serum concentration, pg/ml, mean (SD)** | **Adiponectin serum concentration, μg/l, mean (SD)** | **Leptin serum concentration, μg/l, mean (SD)** |
| --- | --- | --- | --- | --- |
| **CES-D sum scores (N=1092)** | | | | |
| No clinically relevant depressive symptoms  (CES-D sum score:  0-21) | 1042  (95.42%) | 901.27 (433.49) | 7114.30 (4411,84) | 11.02 (11.69) |
| Clinically relevant depressive symptoms (CES-D sum score:  >21) | 50  (4.58%) | 1032.70 (583.24) | 8674.08 (5033.14) | 15.34 (11.22) |
| **IDS sum scores (N=601)** | | | | |
| No clinically relevant depressive symptoms  (IDS sum score:  0-13) | 484  (80.53%) | 866.28 (410.05) | 7444.25 (4632.98) | 11.12 (11.47) |
| Mild intensity of depressive symptoms (IDS sum score: 14-25) | 103  (17.14%) | 969.21 (666.86) | 8438.75 (5008.79) | 16.04 (14.24) |
| Moderate intensity of depressive symptoms (IDS sum score: 26-38) | 13  (2.16%) | 898.23 (574.91) | 8180.15 (4734.74) | 15.89 (13.69) |
| Severe intensity of depressive symptoms (IDS sum score: 39-48) | 1  (0.17%) | 1075.00 (NA) | 16299.00 (NA) | 21.100 (NA) |
| Extreme intensity of depressive symptoms (IDS sum score: >48) | 0 (0%) | --- | --- | --- |

**Notes:** CES-D: Center for Epidemiological Studies Depression Scale (77,78); IDS: Inventory of Depressive Symptomatology – Self Rating 30-item (37,38); NA: not applicable; SD: standard deviation.

**Table S2: Results of multiple linear regression analyses regarding the association between ghrelin serum levels and IDS sum scores reflecting the intensity of depressive symptoms in participants of the study**

| **Variables** | **Regression coefficient β (95% CI)** | **Stan-dardized ß** | **t** | **p value** |
| --- | --- | --- | --- | --- |
| **IDS sum scores in the total sample (N=601)**  Corrected R^2^ = 0.322; F = 36.59; p < 0.001*** | | | | |
| **Multivariate model^a^** | --- | --- | --- | --- |
| Ghrelin serum levels | -0.00048 (-0.0014;0.00045) | -0.036 | -1.014 | 0.311 |
| Age | 0.095 (0.007;0.183) | 0.072 | 2.110 | **0.035*** |
| Gender | 2.011 (1.044;2.977) | 0.160 | 4.086 | **<0.001 ***** |
| Alcohol consumption | 0.010 (-0.012;0.033) | 0.033 | 0.903 | 0.367 |
| Smoking Status | -0.095 (-0.791;0.601) | -0.010 | -0.268 | 0.789 |
| BMI | 0.112 (0.011;0.213) | 0.077 | 2.170 | **0.030*** |
| Cortisol concentration | 0.00011 (-0.003;0.003) | 0.003 | 0.076 | 0.940 |
| GAD-7 sum score | 1.256 (1.098;1.415) | 0.534 | 15.574 | **<0.001 ***** |
| **IDS sum scores in the subgroup of individuals with clinically relevant intensity of depressive symptoms (IDS sum scores ≥ 14) (n=117)**  Corrected R^2^ = 0.133; F = 3.233; p = 0.002** | | | | |
| **Multivariate model^a^** | --- | --- | --- | --- |
| Ghrelin serum levels | -0.00093 (-0.0024;0.00056) | -0.112 | -1.239 | 0.218 |
| Age | -0.104 (-0.298;0.090) | -0.095 | -1.065 | 0.289 |
| Gender | 0.529 (-1.840;2.898) | 0.048 | 0.443 | 0.659 |
| Alcohol consumption | -0.002 (-0.053;0.049) | -0.009 | -0.091 | 0.928 |
| Smoking Status | -0.553 (-2.273;1.166) | -0.059 | -0.638 | 0.525 |
| BMI | 0.032 (-0.163;0.227) | 0.029 | 0.325 | 0.746 |
| Cortisol concentration | 0.0004 (-0.006;0.007) | 0.011 | 0.122 | 0.903 |
| GAD-7 sum score | 0.701 (0.398;1.004) | 0.426 | 4.585 | **<0.001 ***** |
| **IDS sum scores in the subgroup of individuals without clinically relevant intensity of depressive symptoms (IDS sum scores < 14) (n=484)**  Corrected R^2^ = 0.113; F = 8.688; p < 0.001*** | | | | |
| **Multivariate model^a^** | --- | --- | --- | --- |
| Ghrelin serum levels | -0.0004 (-0.0011;0.00032) | -0.049 | -1.071 | 0.285 |
| Age | 0.092 (0.032;0.151) | 0.131 | 3.019 | **0.003**** |
| Gender | 0.790 (0.139;1.441) | 0.118 | 2.383 | **0.018*** |
| Alcohol consumption | 0.008 (-0.007;0.024) | 0.049 | 1.034 | 0.302 |
| Smoking Status | -0.099 (-0.557;0.359) | -0.019 | -0.423 | 0.672 |
| BMI | 0.020 (-0.052;0.092) | 0.025 | 0.537 | 0.591 |
| Cortisol concentration | -0.001 (-0.003;0.001) | -0.033 | -0.753 | 0.452 |
| GAD-7 sum score | 0.483 (0.349;0.617) | 0.309 | 7.074 | **<0.001 ***** |

**Notes:** b: Regression coefficient; β: Standardized regression coefficient; BMI: Body Mass Index; CI: confidence interval; GAD-7: Generalized Anxiety Disorder 7-item Scale (36,79); IDS: Inventory of Depressive Symptomatology – Self Rating 30-item (37,38); N/n: sample sizes. ^+^ p ≤ 0.10; * p ≤ 0.05; ** p ≤ 0.01; *** p ≤ 0.001.

^a^ All multivariate linear regression models have been adjusted for age, gender, alcohol consumption, smoking status (1 = active smoking; 0 = non-smoking), BMI scores, cortisol concentrations and GAD-7 sum scores.

**Table S3: Results of multiple linear regression analyses regarding the association between adiponectin serum levels and IDS sum scores reflecting the intensity of depressive symptoms in participants of the study**

| **Variables** | **Regression coefficient β (95% CI)** | **Stan-dardized ß** | **t** | **p value** |
| --- | --- | --- | --- | --- |
| **IDS sum scores in the total sample (N=601)**  Corrected R^2^ = 0.322; F = 36.615; p < 0.0001*** | | | | |
| **Multivariate model^a^** | --- | --- | --- | --- |
| Adiponectin serum levels | 0.00006 (-0.000045;0.00016) | 0.042 | 1.086 | 0.278 |
| Age | 0.094 (0.006;0.182) | 0.071 | 2.096 | **0.037*** |
| Gender | 1.665 (0.636;2.695) | 0.132 | 3.178 | **0.002**** |
| Alcohol consumption | 0.010 (-0.013;0.032) | 0.030 | 0.825 | 0.410 |
| Smoking Status | -0.140 (-0.832;0.553) | -0.014 | -0.397 | 0.692 |
| BMI | 0.139 (0.036;0.241) | 0.095 | 2.662 | **0.008**** |
| Cortisol concentration | 0.00022 (-0.003;0.003) | 0.005 | 0.146 | 0.884 |
| GAD-7 sum score | 1.251 (1.093;1.408) | 0.532 | 15.575 | **<0.001 ***** |
| **IDS sum scores in the subgroup of individuals with clinically relevant intensity of depressive symptoms (IDS sum scores ≥ 14) (n=117)**  Corrected R^2^ = 0.122; F = 3.019; p = 0.004** | | | | |
| **Multivariate model^a^** | --- | --- | --- | --- |
| Adiponectin serum levels | 0.000042 (-0.00019;0.0003) | 0.039 | 0.367 | 0.715 |
| Age | -0.112 (-0.307;0.082) | -0.103 | -1.144 | 0.255 |
| Gender | -0.042 (-2.612;2.527) | -0.004 | -0.033 | 0.974 |
| Alcohol consumption | -0.005 (-0.056;0.046) | -0.019 | -0.188 | 0.851 |
| Smoking Status | -0.648 (-2.399;1.103) | -0.070 | -0.734 | 0.465 |
| BMI | 0.062 (-0.140;0.265) | 0.057 | 0.610 | 0.543 |
| Cortisol concentration | 0.00029 (-0.006;0.007) | 0.008 | 0.085 | 0.932 |
| GAD-7 sum score | 0.686 (0.382;0.990) | 0.416 | 4.471 | **<0.001 ***** |
| **IDS sum scores in the subgroup of individuals without clinically relevant intensity of depressive symptoms (IDS sum scores < 14) (n=484)**  Corrected R^2^ = 0.111; F = 8.536; p < 0.0001*** | | | | |
| **Multivariate model^a^** | --- | --- | --- | --- |
| Adiponectin serum levels | 0.00001 (-0.000057;0.000077) | 0.014 | 0.289 | 0.773 |
| Age | 0.092 (0.033;0.152) | 0.133 | 3.044 | **0.002**** |
| Gender | 0.672 (-0.016;1.360) | 0.100 | 1.919 | 0.056 |
| Alcohol consumption | 0.007 (-0.008;0.023) | 0.044 | 0.942 | 0.346 |
| Smoking Status | -0.129 (-0.584;0.326) | -0.025 | -0.559 | 0.577 |
| BMI | 0.033 (-0.040;0.105) | 0.041 | 0.883 | 0.377 |
| Cortisol concentration | -0.001 (-0.003;0.001) | -0.030 | -0.690 | 0.490 |
| GAD-7 sum score | 0.478 (0.344;0.612) | 0.305 | 7.009 | **<0.001 ***** |

**Notes:** b: Regression coefficient; β: Standardized regression coefficient; BMI: Body Mass Index; CI: confidence interval; GAD-7: Generalized Anxiety Disorder 7-item Scale (36,79); IDS: Inventory of Depressive Symptomatology – Self Rating 30-item (37,38); N/n: sample sizes. ^+^ p ≤ 0.10; * p ≤ 0.05; ** p ≤ 0.01; *** p ≤ 0.001.

^a^ All multivariate linear regression models have been adjusted for age, gender, alcohol consumption, smoking status (1 = active smoking; 0 = non-smoking), BMI scores, cortisol concentrations and GAD-7 sum scores.

**Table S4: Results of multiple linear regression analyses regarding the association between leptin serum levels and IDS sum scores reflecting the intensity of depressive symptoms in participants of the study**

| **Variables** | **Regression coefficient β (95% CI)** | **Stan-dardized ß** | **t** | **p value** |
| --- | --- | --- | --- | --- |
| **IDS sum scores in the total sample (N=601)**  Corrected R^2^ = 0.325; F = 37.121; p < 0.0001*** | | | | |
| **Multivariate model^a^** | --- | --- | --- | --- |
| Leptin serum levels | 0.055 (0.00027;0.109) | 0.108 | 1.974 | **0.049*** |
| Age | 0.093 (0.005;0.181) | 0.071 | 2.079 | **0.038*** |
| Gender | 1.201 (0.035;2.367) | 0.095 | 2.023 | **0.044*** |
| Alcohol consumption | 0.011 (-0.012;0.033) | 0.034 | 0.921 | 0.357 |
| Smoking Status | -0.132 (-0.823;0.559) | -0.013 | -0.375 | 0.707 |
| BMI | 0.029 (-0.107;0.166) | 0.020 | 0.420 | 0.675 |
| Cortisol concentration | 0.00022 (-0.003;0.003) | 0.005 | 0.149 | 0.882 |
| GAD-7 sum score | 1.251 (1.094;1.408) | 0.532 | 15.618 | **<0.001 ***** |
| **IDS sum scores in the subgroup of individuals with clinically relevant intensity of depressive symptoms (IDS sum scores ≥ 14) (n=117)**  Corrected R^2^ = 0.126; F = 3.087; p = 0.004** | | | | |
| **Multivariate model^a^** | --- | --- | --- | --- |
| Leptin serum levels | 0.056 (-0.090;0.202) | 0.146 | 0.762 | 0.448 |
| Age | -0.107 (-0.302;0.088) | -0.098 | -1.088 | 0.279 |
| Gender | -0.481 (-3.337;2.374) | -0.044 | -0.334 | 0.739 |
| Alcohol consumption | -0.004 (-0.055;0.048) | -0.014 | -0.137 | 0.891 |
| Smoking Status | -0.493 (-2.239;1.253) | -0.053 | -0.560 | 0.577 |
| BMI | -0.072 (-0.446;0.303) | -0.066 | -0.380 | 0.705 |
| Cortisol concentration | 0.001 (-0.006;0.007) | 0.018 | 0.195 | 0.845 |
| GAD-7 sum score | 0.679 (0.375;0.982) | 0.412 | 4.433 | **<0.001 ***** |
| **IDS sum scores in the subgroup of individuals without clinically relevant intensity of depressive symptoms (IDS sum scores < 14) (n=484)**  Corrected R^2^ = 0.112; F = 8.634; p < 0.0001*** | | | | |
| **Multivariate model^a^** | --- | --- | --- | --- |
| Leptin serum levels | 0.016 (-0.020;0.052) | 0.057 | 0.878 | 0.380 |
| Age | 0.092 (0.032;0.151) | 0.132 | 3.021 | **0.003**** |
| Gender | 0.503 (-0.283;1.290) | 0.075 | 1.258 | 0.209 |
| Alcohol consumption | 0.008 (-0.008;0.023) | 0.045 | 0.971 | 0.332 |
| Smoking Status | -0.135 (-0.590;0.320) | -0.026 | -0.584 | 0.560 |
| BMI | 0.005 (-0.084;0.094) | 0.006 | 0.106 | 0.916 |
| Cortisol concentration | -0.001 (-0.003;0.001) | -0.031 | -0.699 | 0.485 |
| GAD-7 sum score | 0.481 (0.347;0.615) | 0.308 | 7.052 | **<0.001 ***** |

**Notes:** b: Regression coefficient; β: Standardized regression coefficient; BMI: Body Mass Index; CI: confidence interval; GAD-7: Generalized Anxiety Disorder 7-item Scale (36,79); IDS: Inventory of Depressive Symptomatology – Self Rating 30-item (37,38); N/n: sample sizes. ^+^ p ≤ 0.10; * p ≤ 0.05; ** p ≤ 0.01; *** p ≤ 0.001.

^a^ All multivariate linear regression models have been adjusted for age, gender, alcohol consumption, smoking status (1 = active smoking; 0 = non-smoking), BMI scores, cortisol concentrations and GAD-7 sum scores.

**Table S5: Results of multiple linear regression analyses regarding the associations between ghrelin, leptin and adiponectin levels and depression scale sub-scores reflecting changes of weight or appetite and sleep disturbances in participants of the study**

| **Variables^a^** | **Ghrelin** | **Leptin** | **Adiponectin** |
| --- | --- | --- | --- |
| **IDS hyposomnia** | b = -0.00023; 95% CI: -0.00053;  0.000071 β = -0.062; t = -1.495; p = 0.135 (n = 603) | b = 0.006; 95% CI: -0.011;  0.024 β = 0.045; t = 0.694; p = 0.488 (n = 604) | b = 0.000010; 95% CI: -0.000022; 0.000042 β = 0.028; t = 0.609; p = 0.543 (n = 604) |
| **IDS hypersomnia** | b = 0.000010; 95% CI: -0.000074; 0.000094 β = 0.010; t = 0.237; p = 0.812 (n = 608) | b = 0.002; 95% CI: -0.003;  0.006 β = 0.038; t = 0.593; p = 0.554 (n = 608) | b = -0.000005; 95% CI: -0.000014; 0.000004 β = -0.045; t = -0.988; p = 0.323 (n = 608) |
| **IDS hypo-appetite** | b = 0.000071; 95% CI:  0.000008;  0.00014 β = 0.098; t = 2.196; p = 0.029* (n = 549) | b = 0.001; 95% CI: -0.002;  0.005 β = 0.054; t = 0.778; p = 0.437 (n = 549) | b = 0.000008; 95% CI: 0.0000006; 0.000015 β = 0.103; t = 2.132; p = 0.033* (n = 549) |
| **IDS hyper-appetite** | b = 0.000007; 95% CI: -0.000040; 0.000054 β = 0.015; t = 0.292; p = 0.770 (n = 472) | b = 0.001; 95% CI: -0.001; 0.004 β = 0.067; t = 0.880; p = 0.379 (n = 472) | b = 0.000004; 95% CI: - 0.000001; 0.000008 β = 0.082;  t = 1.543; p = 0.124 (n = 472) |
| **IDS decrease of weight** | b = -0.000069; 95% CI: -0.00019; 0.000051 β = -0.051; t = -1.132; p = 0.258 (n = 537) | b = -0.003; 95% CI: -0.010; 0.005 β = -0.049; t = -0.705; p = 0.481 (n = 537) | b = -0.0000001; 95% CI: -0.000013; 0.000013 β = -0.001; t = -0.018; p = 0.985 (n = 537) |
| **IDS increase of weight** | b = -0.000045; 95% CI: -0.00018; 0.000085 β = -0.035; t = -0.682; p = 0.496 (n = 436) | b = -0.002; 95% CI: -0.009; 0.006 β = -0.034; t = -0.450; p = 0.653 (n = 436) | b = 0.000007; 95% CI: -0.000006; 0.000020 β = 0.057; t = 1.024; p = 0.306 (n = 436) |
| **CES-D sleep disturbances** | b = -0.00011; 95% CI: -0.00027; 0.000047 β = -0.038; t = -1.377; p = 0.169 (n = 1092) | b = -0.002; 95% CI: -0.012; 0.007 β = -0.023; t = -0.514; p = 0.607 (n = 1092) | b = 0.000017; 95% CI: -0.0000004; 0.000034 β = 0.058; t = 1.918; p = 0.055^+^ (n = 1092) |
| **CES-D appetite disturbances** | b = -0.000006; 95% CI: -0.00008; 0.000067 β = -0.005; t = -0.165; p = 0.869 (n = 1092) | b = 0.002; 95% CI: -0.002; 0.007 β = 0.045; t = 0.957; p = 0.339 (n = 1092) | b = -0.000003; 95% CI: -0.000011; 0.000005 β = -0.027; t = -0.826; p = 0.409 (n = 1092) |
| **CES-D changes of weight** | b = -0.000076; 95% CI: -0.00022; 0.000065 β = -0.033; t = -1.058; p = 0.290 (n = 1092) | b = -0.00035; 95% CI: -0.009; 0.008 β = -0.004; t = -0.080; p = 0.936  (n = 1092) | b = 0.000002; 95% CI: -0.000013; 0.000018 β = 0.011; t = 0.309; p = 0.757  (n = 1092) |

**Notes:** b: Regression coefficient; β: Standardized regression coefficient; BMI: Body Mass Index; CES-D: Center for Epidemiological Studies Depression Scale (81,82); CI: confidence interval; GAD-7: Generalized Anxiety Disorder 7-item Scale (37,83); IDS: Inventory of Depressive Symptomatology – Self Rating 30-item (38,39); N/n: sample sizes. ^+^ p ≤ 0.10; * p ≤ 0.05.

^a^ All multivariate linear regression models (N=27) have been adjusted for age, sex, alcohol consumption, smoking status (1 = active smoking; 0 = non-smoking), BMI scores, cortisol concentrations and GAD-7 sum scores. The dependent variables were the depression scale sub-scores listed in the first column of Table S5. The corresponding independent variables were ghrelin, leptin and adiponectin serum concentrations. The dependent variables were defined as follows: IDS hyposomnia: sum of the IDS items 1-3; IDS hypersomnia: IDS item 4; IDS hypo-appetite: IDS item 11; IDS hyper-appetite: IDS item 12; IDS decrease of weight: IDS item 13; IDS increase of weight: IDS item 14; CES-D sleep disturbances: sum of the CES-D items 5, 11 and 17; CES-D appetite disturbances: CES-D item 1; CES-D changes of weight: CES-D item 16.
